# Cost of post-weaning multi-systemic wasting syndrome and porcine circovirus type-2 subclinical infection in England – An economic disease model

**DOI:** 10.1016/j.prevetmed.2013.02.010

**Published:** 2013-06-01

**Authors:** Pablo Alarcon, Jonathan Rushton, Barbara Wieland

**Affiliations:** Royal Veterinary College, London AL9 7TA, United Kingdom

**Keywords:** Post-weaning multi-systemic wasting syndrome, Porcine Circovirus type 2 subclinical infection, Enterprise budget analysis, Partial budget analysis, Stochastic economic model

## Abstract

Post-weaning multi-systemic wasting syndrome (PMWS) is a multi-factorial disease with major economic implications for the pig industry worldwide. The present study aimed to assess the economic impact of PMWS and porcine circovirus type 2 (PCV2) subclinical infections (PCV2SI) for farrow-to-finish farms and to estimate the resulting cost to the English pig industry.

A disease model was built to simulate the varying proportions of pigs in a batch that get infected with PCV2 and develop either PMWS, subclinical disease (reduce growth without evident clinical signs) or remain healthy (normal growth and no clinical signs), depending on the farm level PMWS severity. This PMWS severity measure accounted for the level of post-weaning mortality, PMWS morbidity and proportion of PCV2 infected pigs observed on farms. The model generated six outcomes: infected pigs with PMWS that die (PMWS-D); infected pigs with PMWS that recover (PMWS-R); subclinical pigs that die (Sub-D); subclinical pigs that reach slaughter age (Sub-S); healthy pigs sold (H-S); and pigs, infected or non-infected by PCV2, that die due to non-PCV2 related causes (nonPCV2-D). Enterprise and partial budget analyses were used to assess the deficit/profits and the extra costs/extra benefits of a change in disease status, respectively. Results from the economic analysis at pig level were combined with the disease model's estimates of the proportion of different pigs produced at different severity scores to assess the cost of PMWS and subclinical disease at farm level, and these were then extrapolated to estimate costs at national level.

The net profit for a H-S pig was £19.2. The mean loss for a PMWS-D pig was £84.1 (90% CI: 79.6–89.1), £24.5 (90% CI: 15.1–35.4) for a PMWS-R pig, £82.3 (90% CI: 78.1–87.5) for a Sub-D pig, and £8.1 (90% CI: 2.18–15.1) for a Sub-S pig. At farm level, the greatest proportion of negative economic impact was attributed to PCV2 subclinical pigs. The economic impact for the English pig industry for the year 2008, prior to the introduction of PCV2 vaccines, was estimated at £52.6 million per year (90% CI: 34.7–72.0), and approximately £88 million per year during the epidemic period.

This was the first study to use empirical data to model the cost of PMWS/PCV2SI at different farm severity levels. Results from this model will be used to assess the efficiency of different control measures and to provide a decision support tool to farmers and policy makers.

## 1 Introduction

In 1996 post-weaning multi-systemic wasting syndrome (PMWS) was first described based on cases occurring in 1991 in Canada (Harding, 1996). Pigs affected by the disease were aged 8–16 weeks and showed wasting or growth retardation, pallor of the skin, respiratory signs and, occasionally, intermittent diarrhoea (Harding and Clark, 1997). Porcine circovirus type 2 (PCV2) was confirmed to be the necessary pathogen (LeCann et al., 1997; Nayar et al., 1997; Allan et al., 1998). Since then, the disease has been observed in most pig producing countries worldwide. However, it has been shown that the presence of PCV2 alone is not enough to trigger clinical signs. Other factors, such as co-infections or environmental conditions and management practices leading to stress and/or increased infectious pressure are believed to be necessary (Madec et al., 2000). The recent successful implementation of PCV2 vaccinations suggest that a subclinical form of the disease was also highly prevalent across farms, as improvement of productivity was higher than could be expected based on clinical PMWS alone (Kurmann et al., 2011; Young et al., 2011). The criterion for the individual diagnosis of PCV2 subclinical pigs (PCV2SI) includes: (1) decrease of average daily gain with no clear clinical signs, (2) no or minimal histiopathological lesions in tissues and (3) presence of low amount of PCV2 in tissues (Segales, 2011b). Opriessnig et al. (2006) showed that PCV2 subclinical infection decreased the efficacy of PRRS vaccine and later hypothesized that PCV2 subclinical infected pigs could have higher susceptibility to other pathogens (Opriessnig et al., 2007). In order to account for the effects of PCV2 infection in epidemiological studies, an approach to define PMWS severity categories at farm level was recently described (Alarcon et al., 2011b). However, despite the acknowledged importance of subclinical pigs, the quantification and the economic impact of these subclinical pigs has not been investigated yet.

Before PCV2 vaccines were available, PMWS had an enormous impact on the productivity on the most of pig industries worldwide (Gillespie et al., 2009). In Europe, during the epidemic stage from 1996 to around 2004, on farm morbidity rates as high as 50–60% and post-weaning mortality between 4 and 20% were reported (Madec et al., 2000; Segales and Domingo, 2002). As the disease became endemic, middle to late 00s, observed morbidity varied considerably between 1% and 30%, while post-weaning mortality remained an important factor for the diagnosis of PMWS at farm level. In England the average post-weaning mortality for non/slightly, moderately and highly PMWS affected farms was 3.7%, 7.3% and 13.5% respectively (Alarcon et al., 2011b). However, despite the major impact of the disease in the industry, no structured economic analysis or disease impact assessment has investigated the economic importance of PMWS. Very few studies have provided an estimation of the cost of this disease. For example, in the United Kingdom the cost of PMWS was estimated at £35 million per year based on crude numbers of pigs dead and not sent to slaughter (Armstrong and Bishop, 2004). Using the same approach and with data extrapolated from the Netherlands, with a 4% reduction in pigs slaughtered due to PMWS, the cost of the disease for the European Union was estimated to be between €562 and €900 million per year (Tucker, 2006). These estimations were only done at industry level for the epidemic period and did not consider the full cost and the complexity of this disease, as only the revenue missed due to PMWS dead pigs were calculated and the costs and losses suffered from subclinical pigs were not included. Recently, a study based on PCV2 vaccination trials, investigated the cost-effectiveness of vaccination in farms affected by porcine circovirus associated disease (PCVAD). The economic model was based on the fact that an infected herd loses 2.5 kg per pig produced plus 0.5 kg per 1% additional mortality due to PCVAD (Burch, 2010). Although this model accounted for losses from subclinical pigs, clinical pigs dying from the disease and clinical pig not dying, only the revenue from the kg missed to produced were considered and no difference in cost between clinical and subclinical infection was obtained.

Assessment of the economic burden of PMWS and PCV2SI is essential to investigate and identify the most cost-effective control strategies and to prioritize the use of scarce resources. In an endemic situation, severity of PMWS and its associated costs can vary considerably across farms (Alarcon et al., 2011b). This indicates a variability of disease importance and that different control measures will differ in efficiency. Control measures that are economically efficient in some situations might not be economically efficient in others situations. Therefore, understanding the economic impact of PMWS and PCV2SI is the necessary and essential first step to optimize control of these diseases. The aim of the current study was to develop an economic model to estimate disease losses at farm level for different PMWS severities. In addition, the study also aims to differentiate between costs and losses from clinical and subclinical pigs.

## 2 Materials and methods

An epidemiological disease model was built to simulate the disease events occurring in a farm at different PMWS severity levels. Economic costs and benefits were calculated separately for the different categories of infected, diseased and healthy pigs. These were then applied to the epidemiological model in order to obtain the overall economic impact of PMWS and PCV2SI. Four data sources were used to build the epidemiological and the economic model:

• Cross-sectional study of 147 English farms carried out in 2008/2009 (CS-2008): Farms in this study were recruited through the PCV2 vaccination programme launch by BPEX in April 2008. However, in order to ensure representativeness of the sample, several farms less affected by PMWS were recruited through veterinary practitioners. All farms were PCV2 unvaccinated at the time of the visit. In each farm 20 blood samples (6 weaners, 6 growers, 6 finishers and 2 sows) were collected and tested for PCV2 by PCR. In addition, data on production performance and on six PMWS morbidity variables were collected (Alarcon et al., 2011b).
• Longitudinal study conducted between 1999 and 2001 (L-2001): This study was conducted in a commercial farm with research facilities in the United Kingdom; nine batches of pigs (*n* = 1080 pigs) were followed over time in an experimental study that tried to assess the impact of different air environment conditions in the health and growth of the pigs (Wathes et al., 2004). Each batch was composed of 120 weaners that were allocated to 5 rooms (24 pigs per room) with different environmental conditions. Over 5 weeks pig weights and feed intake were monitored at animal and pen level, respectively. In addition, blood samples from 371 pigs were available for testing for PCV2 antigen through PCR. Halfway through the experiment the farm was hit by an outbreak of PMWS and affected batches 7–9 (Wieland et al., 2012). Subsequently data of PMWS pigs and PCV2 infected pigs of batches 8 and 9 were collected and provided the basis to derive feed conversion ratios (FCR) and growth rates.
• Farmer opinion survey conducted in 2011 (FO-2011): Twenty farmers were visited after the first version of the epidemiological and economic model was built. Farms were selected randomly from the BPEX PCV2 vaccination programme database. Model uncertainties and assumptions were addressed through farmer's interview and using open and closed questions, i.e. data on PMWS fatality rate, veterinary and labour cost associated with PMWS, and possible slaughterhouse penalty on carcasses from PMWS recovered pigs were collected.
• UK pig industry benchmarking data: Collected for the years 2009 and 2010 (Bench09 and Bench10) for the baseline construction of the model (Anonymous, 2010, 2011). These data were collected by BPEX from farms using the Agrosoft recording software (Agrosoft Ltd., Yew Tree Farm, United Kingdom). For the analysis of the historical economic impact of the disease, some parameters in the benchmarking data from the year 2001–2008 (Bench1 to Bench08) were used to adapt the economic model to each year economic situation (i.e. pig prices, feed prices, etcetera).

### 2.1 PMWS severity case definition

A PMWS severity score was obtained using data from the CS-2008 study with a method described in Alarcon et al. (2011b). Briefly, in a first step, six PMWS morbidity variables were summarised into two factors: morbidity factor 1 (MF1, morbidity observed in weaners and growers), and morbidity factor 2 (morbidity observed in finishers). Subsequently, a farm level PMWS severity score was obtained by combining data on PCV2 PCR results, post-weaning mortality and MF1, using principal component analysis. The derived PMWS severity score ranged between 0 and 10. Farms that scored lower than 4 were considered to be slightly affected; farms with scores higher than 6.5 were classified as highly affected; and farms that scored between 4 and 6.5 were considered as moderately affected by PMWS. In the cross-sectional study, from the 110 farms with complete and reliable data, 27, 58 and 25 farms were identified as ‘Non/Slightly’, ‘Moderately’ or ‘Highly’ affected by PMWS, respectively.

### 2.2 PMWS within-farm epidemiological model

**Fig. 1:**
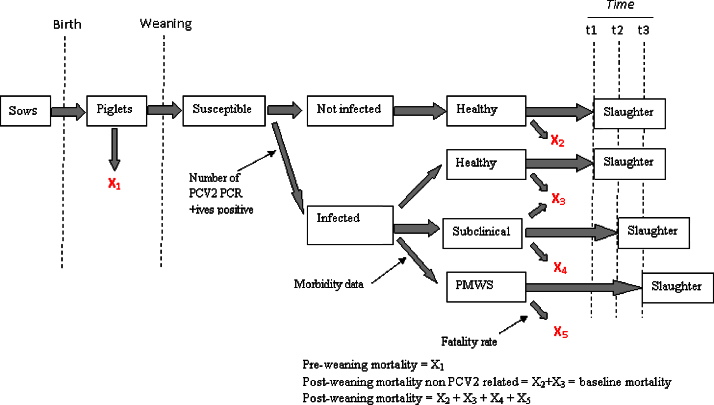
Structure of epidemiological within-farm PMWS disease model.

The epidemiological model assumed that PCV2 infection in a pig could lead to (1) typical PMWS clinical signs, (2) to reduced growth but with no evident clinical sign or (3) to normal growth and no clinical sign (Fig. 1). The latter type of pigs were assumed to not have any cost or economic impact to the farmer. Therefore, for simplicity, in this manuscript ‘subclinical pigs’ are pigs that are infected by PCV2, have reduced growth rates, but no evident clinical signs. This definition of ‘subclinical pigs’ was suggested by Segales (2011b). The definitions and acronyms of the different type of pigs used in this study can be found in Table 1. The proportion of each type of pig in a batch depends on the PCV2 prevalence, PMWS morbidity and the post-weaning mortality of the farm, and therefore on the PMWS severity of the farm.

#### 2.2.1 Model parameters and data fitting for the epidemiological model

##### 2.2.1.1 Time to slaughter (*t*1, *t*2 and *t*3), growth rates and reduction in daily feed consumption

The reference parameters used for healthy pigs were obtained from the English pig industry benchmarking data 2010. In order to get a realistic estimation for values of a healthy pig, only data from the top third farms were used: a healthy pig was weaned at 28 days with a liveweight of 8.1 kg, remained 140 days in feed (*t*1) and was sent to slaughter with at a live weight of 102.6 kg, equal to an average daily gain (ADG) of 0.675 kg/day.

The growth rate parameters used for diseased pigs were derived from data from the two batches of pigs that had PMWS in the L-2001 study. Pigs in this experiment were divided into three categories: (1) non-PCV2 infected pigs, (2) PCV2 infected pigs not showing PMWS clinical signs and (3) PCV2 infected pigs showing PMWS clinical signs. Significance of differences in growth rates (between weaning and day 41 in the experiment) of the three types was assessed through the use of *t*-tests. Reduction in growth rates for PMWS and other PCV2 infected pigs, compared to non-PCV2 infected pigs, were transformed into percentage (% ADG reduction). These percentages were then applied to the reference parameters used for healthy pigs in order to obtain the model ADG for PMWS and PCV2 subclinical pigs. In the case of PMWS-R, it was assumed that the growth rate after recovering from PMWS clinical condition was the same as the growth rate of a subclinical pig. The time needed for a PMWS-R (*t*3) and Sub-S (*t*2) to reach the required slaughter deadweight were obtained using the calculated growth rates.

To assess the appetite loss of a PMWS pig and the reduction in daily feed consumption of a PCV2SI pig, data from the L-2001 study were used. In a linear regression analysis, using data of batches 8 and 9 only, a significant association (*p*-value < 0.0001, *R*^2^ = 0.60) was obtained between the pen level average daily weight gains (exposure variable) and the pen level average daily feed consumption (DFC) (outcome variable), adjusted for pen mortality. Using the linear regression function, the daily feed consumption rate was predicted for a pen with (1) pigs with ADG equal to the non-PCV2 infected pigs, (2) pigs with ADG equal to the PCV2 infected pigs not showing PMWS symptoms, and (3) pigs with ADG equal to the PCV2 infected pigs showing PMWS symptoms. The differences obtained in DFC between pens were transformed into percentages and represented the appetite loss of PMWS pigs and DFC reduction of subclinical pigs (Appendix 1).

##### 2.2.1.2 Proportion of each type of pig in a batch for a given PMWS severity

In order to assess the proportion of each type of pig at different PMWS severity levels, it was important to determine how mortality, morbidity and number of infected pigs increase with an increase in PMWS severity. Therefore, data on post-weaning mortality and proportion of PCV2 infected pigs collected during the CS-2008 study were fitted to the epidemiological model through non-linear regression analysis and mean values for each variable at each PMWS severity level were obtained. Level of PMWS morbidity (MF1) at each severity was then calculated with the Equation 1, which was derived from Alarcon et al. (2011b). In this case, MF1 is a factor score derived from six PMWS morbidity variables, where the variable ‘percentage of PMWS cases in growers’ presented the highest loading. Therefore, in order to obtain the mean percentage of PMWS cases in a batch at different PMWS severity levels, a non-linear regression was also performed using ‘percentage of PMWS cases in growers’ as the outcome variable and MF1 as the predictive variable. Details of the non-linear regression models are shown in Table 2 in the corresponding variables. Once the mean overall post-weaning mortality, mean PMWS morbidity, and mean percentage of PCV2 infected pigs were obtained for each PMWS severity, the proportion of each type of pig in a batch was calculated as follows:

(1) $$\text{MF}1=\frac{e^{\left(\text{PMWS severity}/4.5\right)}-\text{postweaning mortality}*0.5752-\text{PCV}2\;\text{infected}*0.5259-3.48}{0.6266}$$

• *Percentage of nonPCV2-D*: assumed to be equal to the overall post-weaning mortality obtained in an average PMWS none or slightly affected farm (see assumption 1 in Table 3).
• *Percentage of PMWS-D*: derived from the overall number of PMWS cases and the PMWS fatality rate in each PMWS severity score (Eq. (2) and see fatality rate assumption in Table 3).
  (2) PMWS-D=PMWS morbidity*Fatality rate
• *Percentage of PMWS-R*: represented the remaining PMWS cases (overall number of PMWS cases minus fatalities due to PMWS).
• *Percentage of Sub-D*: equal to the post-weaning mortality that was not due to PMWS-D or nonPCV2-D.
• *Percentage of Sub-S*: calculated using Eq. (3):
  (3) Sub-S=(PCV2 prevalence−PMWS morbidity)*Ω

where *Ω* was the proportion of non-PMWS PCV2 infected pigs that have reduced growth rates (see assumption 3 in Table 3).

### 2.3 PMWS economic model

#### 2.3.1 Individual economic analysis

In order to assess the economic impact of disease on each type of pig (H-S, PMWS-D, PMWS-R, Sub-D and Sub-S) two types of economic analyses were carried out: (1) an enterprise budget analysis (EBA) and (2) a partial budget analysis (PBA). However, it is important to first clarify the differences and the relationship between these two types of analyses.

Rushton (2009) defined EBA as an estimate of the difference between the input cost and the output returns for the production of a unit. Considering that input variables can be divided in variable and fixed costs, an enterprise budget analysis can be expressed with the following equation (Eq. (4)):

(4) Enterprise profits=output−variable costs−fixed costs

EBA indicates the profitability of the pig unit. For this study, it was considered that a farm affected by PMWS will produce six different types of units (H-S, nonPCV2-D, PMWS-D, PMWS-R, Sub-D and Sub-S). An EBA was calculated to indicate the profits or deficit normally obtained for each type of unit produced.

Rushton (2009) described PBA as a “technique used to assess small changes in farming systems, a livestock sector enterprise or an existing organization”. It compares the extra costs and extra benefits of a change to indicate if the change is beneficial or if, on the other hand, the change is disadvantageous. The PBA was conducted using the following equation (Eq. (5)):

(5) Net value=(Cost saved+Extra revenue)−(Extra cost+Revenue forgone)

In this study, we considered that a H-S pig would be the normal unit produced by a farmer free of PMWS. However, when a farm is affected by PMWS, the farm would produce some PMWS-D, PMWS-R, Sub-D and Sub-S pigs instead of some H-S pigs. A PBA was therefore conducted to estimate the extra benefits and extra costs of producing diseased pigs instead of H-S pigs. The net value obtained would represent the real cost of producing each type of diseased pig for the farmer.

Both methods, EBA and PBA, used the same data in different ways, with the exception that fixed costs were not included in the PBA. For example, the revenue obtained by selling the carcass of an H-S pig is estimated in £108.39. In an EBA this value appeared as a ‘revenue’ estimate for H-S pig. In a PBA this value appeared as a ‘revenue forgone’ estimate for a PMWS-D and for a Sub-D. In this study, the data obtained from the EBA was used to perform a PBA in order to estimate the extra (or marginal) benefits and extra (or marginal) costs of producing each type of diseased pig instead of a H-S pig.

For the EBA, the feed cost of a pig was obtained by multiplying the total feed consumption of the pig with the price of grower feed. Total feed consumption of a H-S pig was estimated by multiplying the feed conversion rate (obtained from the 2010 benchmarking data using only the top third farms), with the total weight gain of this pig. Feed consumption of diseased pigs were calculated using the daily feed consumption rate, the corresponding days in feed and taking into account the appetite loss of PMWS pigs or the average reduction in daily feed consumption of subclinical pigs. These two latter parameters were obtained from the analysis of the L-2001 study as described in Section 2.2.1.1 and in Appendix 1.

In the EBA, transport costs, levy payments, insurance and inspection costs were only applied to pigs reaching slaughter. Calculation of the veterinary care and treatment (Vet&Med) cost for PMWS pigs was done using a veterinary multiplication factor, obtained from the FO-2011 study, which was multiplied to the reference Vet&Med cost of a H-S pig (see Table 4 footnote). Other variable costs (Vet&Med for subclinical pigs, and electricity, water and bedding costs for all pigs) were calculated according to a pig's time present on the farm. For the EBA, fixed costs were considered constant for all types of pigs.

#### 2.3.2 Farm level economic analysis

Results from the individual PBAs were combined with the disease model's estimates of the proportion of different pigs produced at different severity scores to assess the total cost of PMWS (PMWS-D + PMWS-R) and PCV2SI (Sub-D + Sub-S) at farm level. The combined PMWS and PCV2SI cost was also calculated for each PMWS severity score. Similarly, results from the individual EBAs were combined to estimate the overall profit at farm level. Cost or profits per sow per year was used as the reference parameter for the farm.

#### 2.3.3 Industry level economic analysis

The economic model was re-run by replacing the values of the economic parameters with data from the 2008 benchmarking analysis. The cost of PMWS was estimated for each farm in the CS-2008 study; as details on PMWS severity and number of sows were known for each of the farms. Knowing that the population fraction of the sample of farms in the CS-2008 study was 10.21%, a model inference was done to assess the overall disease cost of the English pig industry for the year 2008.

In addition, an investigation of the industry cost of PMWS and PCV2SI for the period 2001–2007 was done. Average post-weaning mortality and PMWS morbidity per year for the period 2001–2007 were collected for each farm during the CS-2008 study. Although PCV2 prevalence for these farms and for this period was missing, it was estimated by identifying the most likely PCV2 prevalence for a given combination of post-weaning mortality and PMWS morbidity, through linear regression of 2008 data. By combining these three variables, a pseudo-PMWS severity score was calculated for each farm and for each year using the equations from Alarcon et al. (2011b). In order to reduce the risk of misclassification, the pseudo-PMWS severity score was only calculated for farms that passed the misclassification process described in Alarcon et al. (2011b). Data of average number of sows for each farm and each year were also available from the CS-2008 study. The aggregate number of sows of the farms in the CS-2008 study for each year was used to calculate the population fraction of the sample for the corresponding year. Data on production performance, cost and prices for each year were obtained from the benchmarking results published in the corresponding yearbook (Bench01 to Bench08). The economic model was then run and the total combined PMWS and PCV2SI industry cost for each year for the period 2001–2007 was estimated.

### 2.4 Stochastic simulation and sensitivity analysis

Stochastic simulation was performed using @RISK software for Excel version 5.0 (Palisade corporation, Newfield, New York, USA). Distributions were fitted to variables that had some degree of variability or uncertainty (Table 2), and the model was run with 10,000 iterations. Variables believed to have a high impact on the model were assessed in the sensitivity analysis, where variables were changed 10% upwards and downwards from their baseline value. Mean was chosen as reference when the variable output was normally distributed. If variable output was non parametric, the median was selected.

## 3 Results

### 3.1 Epidemiological model

Analysis of the L-2001 data identified that PCV2 infected pigs that developed PMWS clinical signs had a 26% reduction in average daily gain compared to non-PCV2 infected pigs (*p*-value < 0.01). Also, PCV2 infected pigs that did not show PMWS clinical signs, had a significantly lower ADG than non-PCV2 infected pigs (16% ADG reduction, *p*-value < 0.01). Therefore it was estimated that PMWS-R pigs require an extra 33.3 days on the farm to reach the slaughter weight of 102.6 kg, and a Sub-S pig needs 26.7 extra days. Furthermore, it was observed that 73% of the PCV2 infected pigs that did not show PMWS clinical signs had an ADG below the lower 95% confidence interval level of an H-S pig. The parameter values needed for the epidemiological and economic model are summarised in Table 2.

**Fig. 2:**
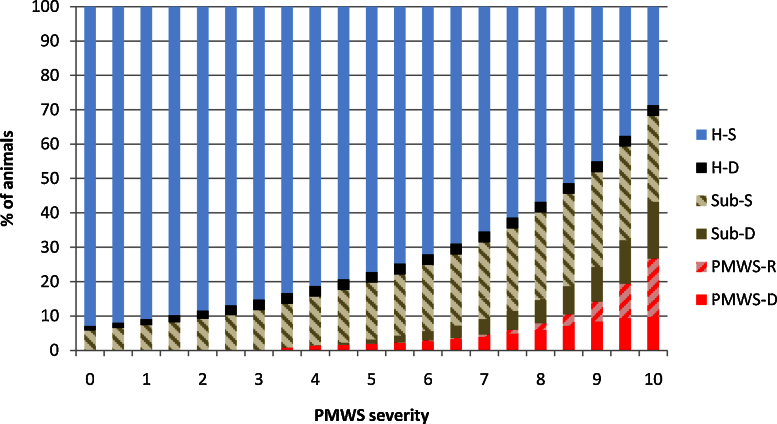
Percentages of each type of pig in a batch at different PMWS severity levels. Where: H-S are healthy pigs with normal growth; PMWS-D are PMWS pigs that die due to the disease; PMWS-R are PMWS pigs that recover from the disease; Sub-D are PCV2 infected pigs with slow growth, no clear PMWS clinical sign and that die; Sub-S are PCV2 infected pigs with slow growth, no clear PMWS clinical sign and that survive; and nonPCV2-D are pigs that die due to non PCV2 related causes.

Outline and results of the epidemiological model after data fitting are presented in Figs. 1 and 2, respectively. The model found that PCV2 subclinical pigs are present at any PMWS severity category, as PCV2 prevalence of slightly affected farms never reaches zero. Further, at all severity levels the number of PCV2 subclinical pigs (Sub-d + Sub-S) was always higher than the number of PMWS pigs (PMWS-D + PMWS-R). The use of exponential functions to fit the data is reflected in the number of H-S pigs decreasing exponentially as severity increases. The proportion of H-S pigs in an average slightly, moderately and highly affected farm was 85.9%, 74.9% and 61.21% respectively.

### 3.2 PMWS economic model

#### 3.2.1 Individual economic analysis

Results from the EBAs are summarised in Table 4. The EBA analysis showed that a farmer has a mean deficit of £-61.6 (90% CI: −67.0 to −57.5) for each PMWS-D pig, £-0.7 (90% CI: −10.6 to 7.9) for each PMWS-R pig, and £-60.6 (90% CI: −65.3 to −56.0) for each Sub-D pig, but will be able to make a profit of £14.9 (90% CI: 11.2–18.1) for each Sub-S pig.

Results from the PBAs, which compared the extra cost and extra benefit of producing a diseased pig instead of a H-S pig, are summarised in Table 5. The PBA shows a marked negative net value for those pigs that died (PMWS-D and Sub-D). The mean net value per PMWS-D pig were £-84.1 (90% CI: −89.1 to −79.6), £-24.6 (90% CI: −35.4 to −15.1) for a PMWS-R pig, £-82.7 (90% CI: −87.5 to −78.1) a Sub-D pig, and £-8.1 (90% CI: −15.1 to −2.2) for a Sub-S pig.

#### 3.2.2 Farm level economic analysis

**Fig. 3:**
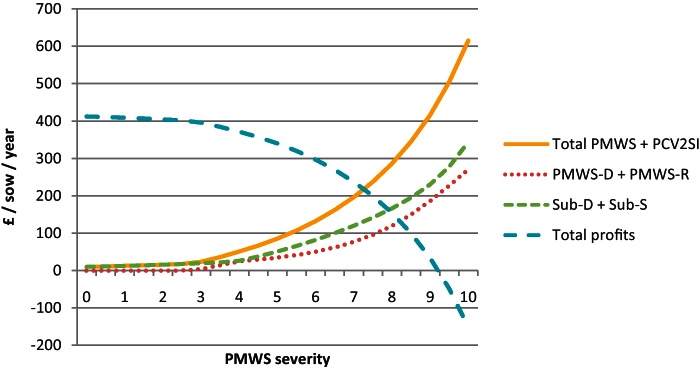
Cost of PMWS and PCV2 subclinical infection (PCV2SI) at farm level and at different PMWS severity levels using 2010 data (deterministic model output). Where: PMWS-D are PMWS pigs that die due to the disease; PMWS-R are PMWS pigs that recover from the disease; Sub-D are PCV2 infected pigs with slow growth, no clear PMWS clinical sign and that die; and Sub-S are PCV2 infected pigs with slow growth, no clear clinical sign and that survive.

**Fig. 4:**
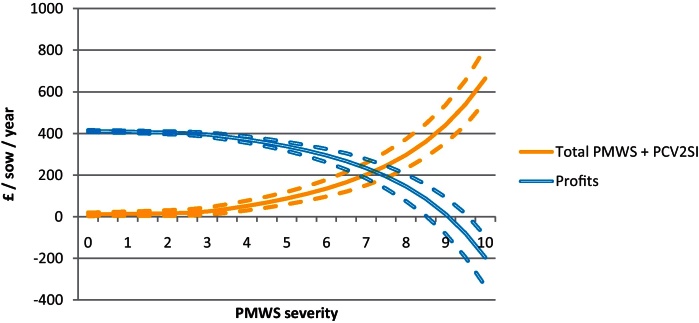
Cost of PMWS and PCV2 subclinical infection (PCV2SI) at farm level and at different PMWS severity levels using 2010 data (stochastic model output). Straight lines shows the mean values and the dotted lines correspond to the 90% confidence intervals.

By combining the results from the individual economic analysis (PBAs) with the proportion of each type of pig at different severities, the overall farm cost of PMWS and PCV2SI for a farm was estimated (Figs. 3 and 4). At farm level, the cost due to PCV2SI was higher than the cost of PMWS pigs at all severity levels. The mean of the combined PMWS and PCV2SI cost for an average slightly, moderately and highly affected farm was £-21.0 (90% CI: −38.9 to −6.3), £-107.5 (90% CI: −142.8 to −75.1) and £-244.7 (90% CI: −313.2 to −179.0) per sow per year, respectively. The profitability of a farm became negative when PMWS severity score was nine (90% CI: 8.5–9.5).

#### 3.2.3 Economic analysis at industry level

**Fig. 5:**
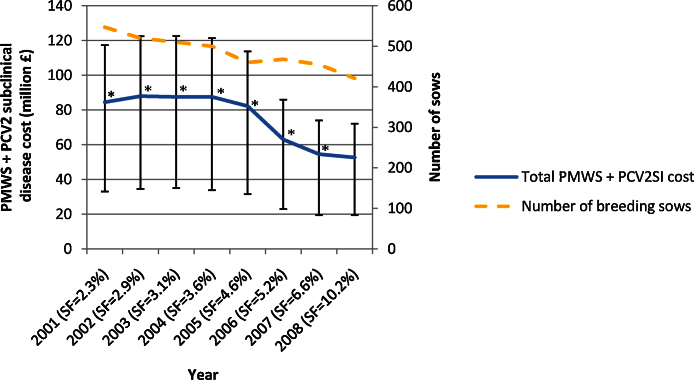
Total combined PMWS and PCV2SI cost for the whole English pig industry from 2001 to 2008. (*) Based on pseudo-PMWS severity estimations. Benchmarking data for the years 2001 to 2008 was used for the following parameters: (1) litters per sow per year, (2) piglets born alive per litter, (3) pre-weaning mortality, (4) sow feed price per tonne, (5) grower feed price per tonne, (6) Vet & Med cost per pig, (6) Electricity cost per pig, (7) Water cost per pig, (8) Straw & Bedding cost per pig, (9) transport cost per pig, (10) miscellaneous or LII cost per pig, (11) labour cost per pig, (12) building cost per pig, (13) equipment cost per pig, (14) other fixed cost per pig, (15) deadweight average price per kg and (16) number of breeding sows.

Model inference indicated that the total combined PMWS and PCV2SI cost for the English pig industry for the year 2008, before implementation of PCV2 vaccination, was £55.7 million (90% CI: 37.8–77.1). Details of the total cost of PMWS and PCV2SI for previous years were estimated using PMWS pseudo-severities and are summarised in Fig. 5. The overall combined PMWS/PCV2SI cost for the period 2001–2007 was £551.1 million (90% CI: 503.5–598.6).

### 3.3 Sensitivity analysis

**Fig. 6:**
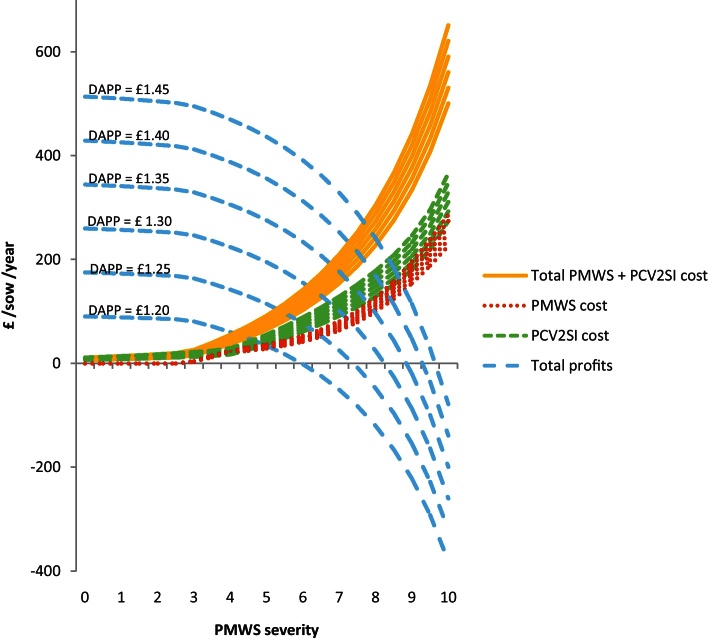
Costs of PMWS, cost of PCV2SI and profitability of the farm at different pig deadweight kilogram prices (DAPP) values. The corresponding ‘DAPP’ is written above its ‘total profit’ line.

Sensitivity analysis indicated that the most influential variables in the model were, in order of importance: (1) post-weaning mortality regression coefficient (total farm cost for an average highly affected farm (TC_H_) ranged between £199.4 and £297.0/sow/year), (2) price per deadweight kg (TC_H_ range = £208.3–283.4/sow/year), (3) average daily gain of a H-S pig (TC_H_ range = £212.1–279.5/sow/year), (4) number of piglets born alive per sow per year (TC_H_ range = £219.8–271.9/sow/year), (4) litters per sow per year (TC_H_ range = £220.2–271.5/sow/year), (5) feed consume per H-S pig (TC_H_ range = £236.1–255.6/sow/year), (6) grower feed price per tonne (TC_H_ range = £236.1–255.7/sow/year), (7) percentage reduction in average daily gain in subclinical pigs (TC_H_ range = £234.9–251.3/sow/year), (8) PCV2 prevalence regression coefficient (TC_H_ range = £238.5–252.6/sow/year), (9) pre-weaning mortality (TC_H_ range = £242.1–249.6/sow/year) and (10) reduction in feed consumption of PCV2 subclinical pigs (TC_H_ range = £242.5–249.2/sow/year). Due to the usual high variability of the price per kilogram deadweight (DAPP) and the fact this was the second most important variable identified in the sensitivity analysis; further analysis was done by running the model with different values of DAPP. Results showed that profitability of the farm was the outcome most affected (Fig. 6).

## 4 Discussion

Post-weaning multi-systemic wasting syndrome is probably one of the most economically damaging diseases for the pig industry worldwide in the last 15 years. To our knowledge this is the first study that uses empirical data, at farm and animal level, to estimate the economic impact of PMWS and PCV2SI. The results indicate the extent of the economic impact of this disease in farms with different PMWS severities and the historical economic impact for the whole English pig industry, which was found to be higher than previously estimated (Armstrong and Bishop, 2004). This is mainly because previous studies did not consider the losses caused by subclinical infection, which economic importance was clearly highlighted in our study. Although the model was based on several assumptions, most of these critical points were addressed through the stochastic simulation.

The approach used for the economic model accounted for the epidemiological complexity of PMWS. In an endemic situation where severity of the disease varies highly between farms, the PMWS severity score proved useful tool to capture this variability. The three components of this severity score increased exponentially, which was the reason why exponential equations were used to fit the data to the model. However, the variability of each of the components within a PMWS severity score was higher as the severity increases, indicating that different PMWS disease scenarios were present in highly affected farms. It is also important to mention that only one farm in the cross-sectional study had a PMWS severity score higher than 8.30 and therefore the model outcomes should be considered as less reliable for high severity levels. Nevertheless, it was useful in estimating the cost of PMWS during the epidemic period, where many farms would have had such a high severity scores.

This study indicates the high farm level cost of PMWS and PCV2SI to pig producers, from which the latter represent the major cost. The importance of these PCV2 subclinical pigs is now widely accepted, in particular since the use of PCV2 vaccines improved productivity of farms more than could be expected by eliminating clinical disease only (Kurmann et al., 2011; Segales, 2011a; Young et al., 2011). The results obtained from the longitudinal study (LS-2001) confirmed the impact of this condition on the growth rate of the pigs and provided useful estimates for the economic model. However, the exact proportion of subclinical pigs on the farms from the cross-sectional study (CS-2008) was difficult to estimate and, to our knowledge, no data is present in the existent literature. Therefore, the model operated with the results from the L-2001study which identified that 73% of non-PMWS PCV2 infected pigs developed subclinical conditions (slow growth). However, the current model does not account for the overall cost of PCV2 as other syndromes caused by this pathogen were not accounted for, namely porcine dermatitis and nephropathy syndrome or PCV2 associated reproductive disorder. This is due to the fact that morbidity data collected on the farm only focused on PMWS and not on other PCV2 syndromes. Yet some of the subclinical pigs that die in this model would have died due to other PCV2 syndromes, and therefore were accounted for to some degree.

Another important factor believed to trigger onset of PMWS is co-infection with other pathogens (Krakowka et al., 2000; Pogranichniy et al., 2002; Opriessnig et al., 2004; Wellenberg et al., 2004; Dorr et al., 2007; Alarcon et al., 2011a). These other pathogens will also have more opportunity to cause harm as the number of subclinical pigs, and therewith pigs with increase susceptibility, increases. This might explain the exponential rise of death of these pigs as PMWS severity increases. Because these deaths might be due to the higher susceptibility caused by PCV2 infection, their economic impact were accounted in the model and attributed to PCV2 subclinical pigs. On the other hand, farms with low PMWS severity scores are likely to be free of other major pathogens, similar to high health farms. Although some of the costs due to other diseases might have been involuntarily accounted for, the model is believed to mainly represent the economic impact of the increase in PMWS severity in a farm.

The total estimated PMWS impact for each year during the epidemic phase (2001–2004) was approximately £88 million, a higher value than previous estimations which did not cover the overall impacts of the disease (Armstrong and Bishop, 2004). This new cost estimate and the figure related to the aggregated cost of the disease for the period 2001–2007 are important to contextualize the amount of research and intervention done on this disease during this period, and to understand the economic impact that novel diseases could have in an industry. Although there were fewer farms affected in the epidemic years than during endemic years, their PMWS severity score was higher. The pig population in England was also higher during the epidemic years, which further contributed to the increased cost of the disease. However, the sampling population fraction of the farms with reliable data for these years was low (2–7%) and representativeness is questionable. Moreover, because of the lack of serum samples for the period 2001–2007, only pseudo-severities could be calculated. Therefore, although these historical economic results may provide a closer understanding to the real cost of PMWS for the epidemic years than previous studies, these should be interpreted with caution. On the other hand, the data obtained from 2008 cross-sectional study is believed to be representative of the English pig industry. Although many of the farms in the CS-2008 study were recruited through the BPEX PCV2 vaccination scheme, and were therefore more likely to be moderately or highly affected by PMWS, the recruitment of other farms not participating in this scheme, through veterinary practitioners, reduced the farm selection bias and therefore increased the representativeness of the sample. Further the industry sample fraction of the 2008 data (10.2%) and the spatial distribution of the farms that participated in the CS-2008 study (data not shown) indicate a good representation of the English pig industry. Nonetheless, it is important to note that the industry analysis performed in this study did not account for price fluctuations in relation to supply and demand. In theory, the increase in production costs and the decrease in productivity due to PMWS/PCV2SI would have caused prices to rise. This effect would be felt unevenly across the industry depending on whether an individual operation was affected by PCV2 or not. Non-affected operations would benefit from the price increases and increased profitability, somewhat offsetting the losses and cost increases incurred by affected operations. Consumers would also be negatively impacted by the changes in price. Overall, it is possible that an overestimation of the cost of the disease at industry level might have occurred.

The economic tools used for this analysis, enterprise budget analysis and partial budget analysis, are widely used in animal health economics. Both analyses provided different types of information that are relevant to producers. The EBA indicates what is the actual profit or deficit of producing each type of pig. This information is relevant as it helps to understand the impact of the disease to the economic situation of the farm, such as cash flow effects and overall profitability. On the other hand, PBA indicates the real cost of the disease, as it shows the losses incurred by producing diseased pigs instead of healthy pigs. The economic structure used for the enterprise budget analysis was based on the structure provide by BPEX, the English pig levy payer association, in their pig yearbook journals. However, a proportion of the labour cost can normally be classified as variable cost, when part of the payment is subjected to productivity of the farm or extra hours needed (Rushton, 2009). Yet, due to the uncertainty of the possible variable cost associated with labour in relation to PMWS, further investigation was done during the FO-2011 study. Most of the farmers in this study declared that, although they had to work more due to PMWS, no extra labour fee was normally paid (data not shown). Consequently, labour was maintained as a fixed cost in this study. Nonetheless, further investigation into the opportunity cost due to the extra labour would allow the model to give even more precise estimations of the true cost of PMWS. Similarly, disease impact on the other fixed cost parameters was not accounted in this model, and therefore some underestimation might have occurred in this regard.

Several of the parameters used to assess the cost of disease were based on pig industry benchmarking data. This data only includes farms using Agrosoft recording software and might have lead to some bias in the estimation of some parameters. Using the top third farms benchmarking data to inform some parameters for the growth and feed intake of H-S pigs was based on the assumption that these farms would be more likely to be free of diseases and, therefore, to have values most representative of H-S pig. However, these farms could also have significant differences in management, breed or farm structure compare to average farms. Therefore, some bias might have occurred. Furthermore, fixed costs were assumed to be the same for all farms in the study. The online accessible version of the model however, allows farmers to input their own specific values (e.g. H-S pig production values, fixed costs, etcetera).

Several other biases and limitations need to be considered when interpreting the findings. The most important is related to the PCV2 prevalence estimation at farm level which was obtained through stratified sampling of pigs in the cross-sectional study by age matching of PMWS affected and healthy pigs in each age group. Although the proportion of PCV2 PCR positive pigs provides a good contributing indicator of PMWS severity, its use as PCV2 within-farm prevalence estimate might have resulted in an overestimation or underestimation of the real number of infected pigs on the farm. However, the fact that apparently healthy pigs were also consistently included in the sample may relax somewhat this overestimation, and the combined results, from PMWS and health pigs, may be considered as a reasonable approximation of the real prevalence. This model also assumed that no compensatory growth occurred on PMWS-R pigs after recovering from the disease. The compensatory growth effect in pigs has been described by several authors after an abrupt transition of feed composition and feeding regime given to growing pigs (Heyer and Lebret, 2007). However, to the authors’ knowledge, this effect has not been reported as a sign of recovering from PMWS. Nonetheless, it is possible that an overestimation of the extra costs associated to these pigs might have occurred due to this assumption.

The current study provides a useful decision support tool to illustrate farmers about the costs of a typical production disease (http://www.bpex.org.uk/R-and-D/R-and-D/PMWSEpidemiology.aspx). This economic model further will be used to investigate the efficiency of different control measures through investment appraisal and a scenario analysis.

## Figures and Tables

**Table 1:** Acronyms and definitions for the different types of pigs used in the economic model. It is important to note that a proportion of H-S pigs are also infected by PCV2. However, these pigs were assumed to not have any economic impact for the farmer. Therefore, the acronym ‘PCV2SI’ in this study only refers to the combination of Sub-D and Sub-S.

Type of pigs in the model	Definition	Group classification
PMWS-D	PCV2 infected pig that develops PMWS clinical signs causing death or the necessity of being culled	PMWS pigs
PMWS-R	PCV2 infected pig that develops PMWS clinical signs, but is able to recover, resulting in delayed slaughter (*t*3 − *t*1).	

Sub-D	PCV2 infected pig, that shows no clinical PMWS, but that has a reduced growth rate and increased susceptibility for co-infection with other pathogens, leading to death.	Subclinical pigs (PCV2SI)
Sub-S	PCV2 infected pig that shows no clinical PMWS, but has a reduced growth rate and reduced feed intake. This pig will take longer (*t*2 − *t*1) to reach the required weight at slaughter.	

H-S	Pig, infected or not infected by PCV2, that remains healthy throughout its life, with normal growth and reaches age to slaughter at *t*1.	Healthy pig sold

NonPCV2-D	These pigs can either be non infected by PCV2 or infected by PCV2 but with no PMWS clinical signs. The death of these pigs is not due to PCV2 infection.	Pig that dies due to non-PCV2 related causes

**Table 2:** Parameters for the economic disease model. Where: H-S are healthy pigs with normal growth; PMWS-D are PMWS pigs that die due to the disease; PMWS-R are PMWS pigs that recover from the disease; Sub-D are PCV2 infected pigs with slow growth, no clear PMWS clinical sign and that die; and Sub-S are PCV2 infected pigs with slow growth, no clear PMWS clinical sign and that survive.

Parameters	Symbol	Value	Stochastic		Reference
			Distribution	Range	
**Post-weaning mortality**					
Overall post-weaning mortality	*X*_T_	1.29*exp(0.313*PMWS severity)	Normal	0.27–0.36	Fitted through non-linear regression of CS-2008 data (reg coef. *p* < 0.01, *R*^2^ = 0.88). The 95% confidence interval of the regression coefficient was used to define the stochastic range.
Post-weaning mortality non-PCV2 related (%)	*X*_2+3_	3.08	–	–	Post-weaning mortality at severity of 2.79 (see assumption 7 in Table 3)
Post-weaning mortality attributable to PMWS pigs	*X*_5_	ψ*FR	–	–	[Rule: Cannot be higher than the proportion of PCV2 infected pigs]
PMWS fatality rate (FR)	FR	1.0136*exp(−0.0382*ψ)	–	–	Fitted through non-linear regression of FO-2011 data (reg coef. *p* = 0.17, *R*^2^ = 0.82)
Post-weaning mortality attributable to PCV2 subclinical pigs	*X*_4_	*X*_T_ − *X*_2+3_ − *X*_5_	–	–	[Rule: *X*_4_ cannot be less than zero]

**PMWS morbidities and PCV2 prevalence**			–	–	
Proportion of PCV2 infected pigs	π	0.0982*exp(0.2244*PMWS severity)	Normal	0.18–0.27	Fitted through non-linear regression of CS-2008 data (*p* < 0.01, *R*^2^ = 0.87). The 95% confidence interval of the regression coefficient was used to define the stochastic range.
Percentage of PMWS pigs	*ψ*	0.1127*exp(2.8802*MF1)	Normal	2.61–0.15	Fitted through non-linear regression of CS-2008 data (*p* < 0.01, *R*^2^ = 0.82)). The 95% confidence interval of the regression coefficient was used to define the stochastic range.
Morbidity factor 1	MF1	See Eq. (1)	–	–	
Proportion of PCV2 infected pigs that develop subclinical signs	*Ω*	0.73	Normal	0.27–0.48	Estimated from data of L-2001 (see assumption 3 in Table 3). The 95% confidence interval was used to define the stochastic range.
**Growth rate parameters**					
Average weight at slaughter of a H-S pig (kg)	*W*_*t*1_	102.6	–	–	*T*_1_*ADG_H-S_ + *W*_*t*0_
Average weight at weaning of a H-S pig (kg)	*W*_*t*0_	8.1	–	–	Bench10
Average daily weight gain of a H-S pig (kg/day)	ADG_H-S_	0.675	–	–	Bench10
Percentage reduction in ADG of a Sub-S pig	*ζ*_Sub_	16	Normal	3–29	Estimated from data of L-2001 study. The 95% confidence interval was used to define the stochastic range.
Average daily weight gain of a Sub-S pig (kg/day)	ADG_Sub_	0.567	–	–	ADG_H-S_*(1 − *ζ*_Sub_)
Percentage reduction in ADG of a PMWS-R pig	*ζ*_PMWS_	26	Normal	16–36	Estimated from data of L-2001 study. The 95% confidence interval was used to define the stochastic range.
Average daily weight gain of a PMWS-R pig (kg/day)	ADG_PMWS_	0.500	–	–	ADG_H-S_*(1 − *ζ*_PMWS_)
Average weight at death of a PMWS-D pig (kg)	*W*_PMWSD_	30.37	–	–	*W*_*t*0_ + *T*_PMWSD_*ADG_PMWS_
Average weight at death of a Sub-D pig (kg)	*W*_SubD_	42.12	–	–	*W*_*t*0_ + *T*_SubD_*ADG_Sub_

**Time parameters**					
Average days in feed of a H-S pig (days)	*T*_1_	140	–	–	Bench10
Average days in feed of a Sub-S pig (days)	*T*_2_	166.67	–	–	(*W*_*t*1_ − *W*_*t*0_)/ADG_Sub_ [161 days in the model as explained in assumption 8, Table 3)
Average days in feed of a PMWS-D pig (days)	*T*_PMWSD_	56	Beta pert	42–70	See assumption 6, Table 3
Average days in feed of a Sub-D pig (days)	*T*_SubD_	56	Uniform	50–80	See assumption 6, Table 3
Average days in feed of a PMWS-R pig (days)	*T*_3_	173.81	–	–	((*T*_PMWSD_) + ((*W*_HS_ − *W*_PMWSD_)/ADG_Sub_)

**Breeding parameters**					
Litters per sow per year	LSY	2.2	–	–	Bench10
Pigs born alive per sow per litter	PBSL	11.20	–	–	Bench10
Mortality of pigs born alive (%)	*X*_1_	12.72	–	–	Bench10
Pigs weaned er sow per year	WSY	21.99	–	–	LSY*PBSY*(1 − (*X*_1_/100))
Sow year replacement rate (%)	SRR	49.25	–	–	Bench10
Average number of parities	AP	4.56	–	–	(100/SRR)*LSY
Cost of replacing a gilt (£)	CG	157.62	–	–	Bench09

**Feed parameters**					
Sow feed consumption per year (kg)	FQ_S_	1232	–	–	Bench10
Sow feed price/tonne (£)	FP_S_	162.87	–	–	Bench10
Grower feed price/tonne (£)	FP_G_	202.55	–	–	Bench10
Feed conversion of a H-S pig	FCR_HS_	2.39	–	–	Bench10
Feed consumed per H-S pig (kg)	FQ_HS_	225.85	–	–	FCR_HS_*(*W*_*t*1_ − *W*_*t*0_)
Feed consumed per day per H-S pig (kg)	DFQ_HS_	1.61	–	–	FQ_HS_/*T*_1_
Appetite loss of a PMWS-D and PMWS-R pigs during the clinical stage (%)	AL_PMWS_	17	Linked to *ζ*_PMWS_	Linked to *ζ*_PMWS_	Estimated from data of L-2001 study trough the correlation observed between feed intake and average daily gain at pen level. See Appendix 1.
Reduction in feed consumption by subclinical pigs (%)	AL_Sub_	10	Linked to *ζ*_Sub_	Linked to *ζ*_Sub_	Estimated from data of L-2001 study trough the correlation observed between feed intake and average daily gain at pen level. See Appendix 1.
Feed consumed per PMWS-D pig (kg)	FQ_PMWSD_	79.37	–	–	DFQ_HS_*(1 − (AL_PMWS_/100))*T_PMWSD_
Feed consumed per Sub-D pig (Kg)	FQ_SubD_	104.86	–	–	DFQ_HS_*T_SubD_
Feed consumed per PMWS-R pig (kg)	FQ_PMWSS_	242.31	–	–	FQ_PMWSD_ + (DFQ_HS_*(*T*_3_ − *T*_PMWSD_)
Feed consumed per Sub-S pig (kg)	FQ_SubS_	259.72	–	–	DFQ_HS_*T_2_
**Other parameters**					
Veterinary multiplication factor	VMF	2.74	Beta pert	1–8.06	Average obtained from FO-2011 study. Minimum and maximum values obtained were used to define the stochastic range.
Cost of disposing a dead carcass (£)	DC	6	–	–	As reported by a farmer in the FO-2011 study.
Penalty on PMWS carcass (pp/kg)	PC	8.75	Beta pert	0–35	Average obtained from FO-2011 study. Minimum and maximum values obtained were used to define the stochastic range.
Deadweight average pig price (£/kg)	DAPP	1.39	–	–	Bench10

**Table 3:** Main assumptions made on the epidemiological and economic model. Where: H-S are healthy pigs with normal growth; PMWS-D are PMWS pigs that die due to the disease; PMWS-R are PMWS pigs that recover from the disease; Sub-D are PCV2 infected pigs with slow growth, no clear PMWS clinical sign and that die; and Sub-S are PCV2 infected pigs with slow growth, no clear clinical sign and that survive.

N	Assumptions	Justification
1.	The PMWS fatality rate is not constant. It is assumed to be high (100%) when few cases are observed and decreases when the number of cases increases substantially.	This study assumes that the farmer will try to recover as many pigs as possible when the number of cases is high. The validity of this assumption was investigated with the data collected in the FO-2011 (data not shown). The reduction in fatality rate used for this study was calculated by fitting this data into the model.

2.	Percentage of PCV2 PCR positive pigs was used as PCV2 prevalence for each PMWS severity score.	Due to lack of prevalence data in relation to PMWS severity scores, this proportion was assumed to be a reasonable approximation of the PCV2 prevalence at farm level.

3	73% of non-PMWS PCV2 infected pigs develop subclinical signs (*Ω*). This measure is constant at any PMWS severity score.	This was estimated using the L-2001 study. In that study 73% of infected pigs, not showing PMWS clinical signs, had an ADG below the lower 95% confidence interval level of non-PCV2 infected pigs.

5	A PMWS-R pig after recovering has an ADG equal to the PCV2 subclinical pig.	The retardation in growth suffered during the clinical stage makes the pig unable to grow at a normal healthy speed when they recover from the disease.

6	A PMWS-R pig recovers and a PMWS-D pig dies 56 days after weaning; a Sub-D pig dies at 56 days in feed.	The window of PMWS is between 8 and 16 weeks of age, therefore 12 weeks was chosen as the average age of death or recovery of a PMWS pig. To adjust for uncertainty the age at death/recovery of a PMWS pig was varied by 2 weeks in the stochastic model. The age of death of a Sub-D was also varied in the stochastic model to account for uncertainty (see Table 2)

7	The non-PCV2 related post-weaning mortality (*X*_2_) is assumed to be equal to the level of post-weaning mortality observed in an average slightly affected farm with a PMWS score of 2.79	Farms with PMWS scores below the average slightly affected severity score (2.79) are assumed to be free of PMWS or with insignificant morbidity levels. Although few subclinical pigs may be present, no death due to PCV2 occurs.

8	Sub-S pigs are not detected by the farmer or the vet and therefore are treated like H-S pigs, but they need more time to reach the slaughter weight.	Subclinical per se is not easy to detect by farmers or vets, as no clinical signs are shown. Although these pigs are more susceptible to other pathogens, it was assumed that the condition is not detected by the farmer or the vet.

9	Fixed costs were assumed to be equal to all the farms in industry level analysis.	The fixed costs used were obtained from the English pig industry benchmarking data and represent the average fixed costs of farms.

**Table 4:** Results from the enterprise budget analyses. Mean values from the stochastic model are shown. Where: H-S are healthy pigs with normal growth; PMWS-D are PMWS pigs that die due to the disease; PMWS-R are PMWS pigs that recover from the disease; Sub-D are PCV2 infected pigs with slow growth, no clear PMWS clinical sign and that die; and Sub-S are PCV2 infected pigs with slow growth, no clear PMWS clinical sign and that survive.

		Value (£)				
		H-S	PMWS-D	PMWS-R	Sub-D	Sub-S
Revenue	Total deadweight sold^a^	108.39	0	99.29	0	108.39
	Depreciation of sow^b^	−3.54	−3.54	−3.54	−3.54	−3.54
Variable costs	Sow feed^c^	−9.12	−9.12	−9.12	−9.12	−9.12
	Pig feed^d^	−45.75	−15.28	−49.81	−19.08	−49.05
	Vet&Med cost^e^	−1.87	−6.24	−7.82	−0.87	−2.24
	Transport^f^	−2.34	0	−2.34	0	−2.34
	Carcass disposal	0	−6	0	−6	0
	Electricity^g^	−2.12	−0.85	−2.64	−0.98	−2.54
	Water^g^	−0.60	−0.24	−0.75	−0.28	−0.72
	Straw & bedding^g^	−0.43	−0.17	−0.53	−0.20	−0.52
	Levy, insurance, inspection^h^	−2.90	0	−2.90	0	−2.90

Gross margin^j^		39.72	−41.94	19.85	−40.07	35.42

Fixed costs	Labour^i^	−10.83	−10.83	−10.83	−10.83	−10.83
	Building^i^	−3.47	−3.47	−3.47	−3.47	−3.47
	Equipment^i^	−3.51	−3.51	−3.51	−3.51	−3.51
	Other fixed costs^i^	−2.68	−2.68	−2.68	−2.68	−2.68

Net margin^j^		**19.23**	−**61.64**	−**0.65**	−**60.57**	**14.92**

a Assuming that the deadweight of a pig is 76% of its live weight (English et al., 1988). The carcass value of a pig was then obtained by multiplying the DAPP to the carcass weight at slaughter. For PMWS-R pigs a penalty cost (PC, see Table 2) was subtracted for each deadweight kg at slaughter.

b Depreciation of a sow was calculated as: CG/(AP*WSY).

c Sow feed cost was calculated as: (FQ_S_*FP_S_)/WSY.

d Feed cost was estimated multiplying the quantity of feed consumed by each type of pig with FP_G_.

e The cost of Veterinary care and Medical treatment (Vet&Med) was 2.4 pp per kg deadweight (Bench10). Therefore, the cost for a H-S pig was obtained by multiplying this with the total deadweight at slaughter. The Vet&Med cost for PMWS-D was calculated by multiplying the value obtained for an H-S pig by the VMF. The Vet&Med cost for PMWS-R was derived by summing two costs: Vet&Med cost of a PMWS-D pig + the equivalent Vet&Med cost for the time spent in the farm after recovering from disease (extrapolated from the H-S cost). The Vet&Med cost for a Sub-D and a Sub-S pig was obtained by extrapolating the cost obtained for a H-S pig according to their days in feed.

f Obtained from Bench09 for H-S, PMWS-R and Sub-S pigs.

g Reference cost for a H-S pig was obtained from Bench09. Costs for diseased animals were obtained by extrapolating according to their days in feed.

h Reference cost for a H-S pig was obtained from Bench10. Only pigs sent to slaughter are subject to this cost.

i The cost of labour was £13.9 per kg deadweight (Bench10). This was multiplied by the deadweight of a H-S pig. Building, equipment and other costs were obtained from Bench09. Fix costs were assumed constant for each type of pig.

j Gross and net margin values in this table correspond to the mean values obtained after the stochastic model. Therefore, the values will not correspond to the exact calculations from the above values.

**Table 5:** Results from the partial budget analyses. Mean values from the stochastic model are shown. Where: H-S are healthy pigs with normal growth; PMWS-D are PMWS pigs that die due to the disease; PMWS-R are PMWS pigs that recover from the disease; Sub-D are PCV2 infected pigs with slow growth, no clear PMWS clinical sign and that die; and Sub-S are PCV2 infected pigs with slow growth, no clear PMWS clinical sign and that survives.

		Value (£)			
		PMWS-D	PMWS-R	Sub-D	Sub-S
Extra cost	Extra pig feed^a^	0	−4.06	0	−3.31
	Extra Vet&Med costs^b^	−4.37	−5.95	0	−0.37
	Extra electricity^b^	0	−0.52	0	−0.42
	Extra water^b^	0	−0.15	0	−0.12
	Extra straw & bedding^b^	0	−0.10	0	−0.09
	Extra levy, insurance, inspection costs^b^	0	0	0	0
	Carcass disposal^b^	−6	0	−6	0
Revenue forgone	Deadweight not sold	−108.39	0	−108.39	0
	Loss in carcass quality^c^	0	−9.10	0	0
	Profit lost due to miss-used space^d^	0	−4.58	0	−3.66

**Total extra costs**		−**112.76**	−**24.34**	−**114.39**	−**13.86**

Costs saved	Pig feed saved^a^	30.47	0	26.66	0
	Vet&Med costs saved^b^	0	0	1.00	0
	Electricity saved^b^	1.27	0	1.14	0
	Water saved^b^	0.36	0	0.32	0
	Straw & bedding saved^b^	0.26	0	0.23	0
	Extra levy, insurance, inspection costs saved^b^	2.90	0	2.90	0
	Transport saved^b^	2.34	0	2.34	0
Extra revenue	None	−	−	−	−

**Total benefits**		**37.60**	**0**	**32.43**	**0**
**Net total**^e^		−**84.06**	−**24.55**	−**82.69**	−**8.11**

a Differences in feed consumed, compared to a H-S pig, were multiplied by FP_G_.

b Difference in cost compared to a H-S pig.

c Carcass weight of a PMWS-R pig at slaughter was multiplied by PC (see Table 4).

d Calculated only for PMWS-R and Sub-S pigs because of their extra days in feed on the farm. The space they occupied could have been used to improve the productivity of the farm. To account for this loss of potential, the profits obtained for each H-S pig produced in 140 days was extrapolated to the extra days in feed that these disease pigs remained on the farm.

e Net margin values in this table correspond to the mean values obtained after the stochastic model. Therefore, the values will not correspond to the exact calculations from the above values.

## References

[bib0005] Alarcon P., Velasova M., Mastin A., Nevel A., Stark K.D., Wieland B. (2011). Farm level risk factors associated with severity of post-weaning multi-systemic wasting syndrome. Prev. Vet. Med..

[bib0010] Alarcon P., Velasova M., Werling D., Stärk K.D.C., Chang Y., Nevel A., Pfeiffer D.U., Wieland B. (2011). Assessment and quantification of post-weaning multi-systemic wasting syndrome severity at farm level. Prev. Vet. Med..

[bib0015] Allan G., Meehan B., Todd D., Kennedy S., McNeilly F., Ellis J., Clark E.G., Harding J., Espuna E., Botner A., Charreyre C. (1998). Novel porcine circoviruses from pigs with wasting disease syndromes. Vet. Rec..

[bib0020] Anonymous, 2010. Pig Yearbook 2010. BPEX.

[bib0025] Anonymous, 2011. Pig Yearbook 2011. BPEX.

[bib0030] Armstrong D., Bishop S.C. (2004). Does genetics or litters effect influence mortality in PMWS. Proceedings of the 18th International Pig Veterinary Society (IPVS) Congress.

[bib0035] Burch D. (2010). The Effect of PCV2 on Liveweight and Mortality. http://www.octagon-services.co.uk/articles/PCV2effect.pdf.

[bib0040] Dorr P.M., Baker R.B., Almond G.W., Wayne S.R., Gebreyes W.A. (2007). Epidemiologic assessment of porcine circovirus type 2 coinfection with other pathogens in swine. J. Am. Vet. Med. Assoc..

[bib0155] English P.R., Fowler V.R., Baxter S., Smith B. (1988). The Growing and Finishing Pig: Improving Efficiency.

[bib0045] Gillespie J., Opriessnig T., Meng X.J., Pelzer K., Buechner-Maxwell V. (2009). Porcine circovirus type 2 and porcine circovirus-associated disease. J. Vet. Intern. Med..

[bib0050] Harding J., Clark E.G. (1997). Recognizing and diagnosing postweaning multisystemic wasting syndrome (PMWS). J. Swine Health Prod..

[bib0055] Harding J.C. (1996). Post-weaning multisystemic wasting syndrome: preliminary epidemiology and clinical findings. Proceedings of West Can. Association of Swine Practitioners.

[bib0060] Heyer A., Lebret B. (2007). Compensatory growth response in pigs: effects on growth performance, composition of weight gain at carcass and muscle levels, and meat quality. J. Anim. Sci..

[bib0065] Krakowka S., Ellis J.A., Meehan B., Kennedy S., McNeilly F., Allan G. (2000). Viral wasting syndrome of swine: experimental reproduction of postweaning multisystemic wasting syndrome in gnotobiotic swine by coinfection with porcine circovirus 2 and porcine parvovirus. Vet. Pathol..

[bib0070] Kurmann J., Sydler T., Brugnera E., Buergi E., Haessig M., Suter M., Sidler X. (2011). Vaccination of dams increases antibody titer and improves growth parameters in finisher pigs subclinically infected with PCV2. Clin. Vaccine Immunol..

[bib0075] LeCann P., Albina E., Madec F., Cariolet R., Jestin A. (1997). Piglet wasting disease. Vet. Rec..

[bib0080] Madec F., Eveno E., Morvan P., Hamon L., Blanchard P., Cariolet R., Amenna N., Morvan H., Truong C., Mahe D., Albina E., Jestin A. (2000). Post-weaning multisystemic wasting syndrome (PMWS) in pigs in France: clinical observations from follow-up studies on affected farms. Livest. Prod. Sci..

[bib0085] Nayar G.P., Hamel A., Lin L. (1997). Detection and characterization of porcine circovirus associated with postweaning multisystemic wasting syndrome in pigs. Can. Vet. J..

[bib0090] Opriessnig T., McKeown N.E., Harmon K.L., Meng X.J., Halbur P.G. (2006). Porcine circovirus type 2 infection decreases the efficacy of a modified live porcine reproductive and respiratory syndrome virus vaccine. Clin. Vaccine Immunol..

[bib0095] Opriessnig T., Meng X.J., Halbur P.G. (2007). Porcine circovirus type 2 associated disease: update on current terminology, clinical manifestations, pathogenesis, diagnosis, and intervention strategies. J. Vet. Diagn. Invest..

[bib0100] Opriessnig T., Thacker E.L., Yu S., Fenaux M., Meng X.J., Halbur P.G. (2004). Experimental reproduction of postweaning multisystemic wasting syndrome in pigs by dual infection with Mycoplasma hyopneumoniae and porcine circovirus type 2. Vet. Pathol..

[bib0105] Pogranichniy R.M., Yoon K.J., Harms P.A., Sorden S.D., Daniels M. (2002). Case–control study on the association of porcine circovirus type 2 and other swine viral pathogens with postweaning multisystemic wasting syndrome. J. Vet. Diagn. Invest..

[bib0110] Rushton J. (2009). The Economics of Animal Health and Production. http://www.cabi.org/.

[bib0115] Segales J. (2011). The Importance of Porcine Circovirus Type 2 (PCV2) Subclinical Infection. http://www.pig333.com/circovirosis/the-importance-of-porcine-circovirus-type-2-pcv2-subclinical-infecti_4052/.

[bib0120] Segales J. (2011). Porcine circovirus type 2 (PCV2) infections: clinical signs, pathology and laboratory diagnosis. Virus Res..

[bib0125] Segales J., Domingo M. (2002). Postweaning multisystemic wasting syndrome (PMWS) in pigs. A review. Vet. Q..

[bib0130] Tucker A.W. (2006). Porcine multi-systemic wasting syndrome (PMWS): a review. Pig J..

[bib0135] Wathes C.M., Demmers T.G.M., Teer N., White R.P., Taylor L.L., Bland V., Jones P., Armstrong D., Gresham A.C.J., Hartung J., Chennells D., Done S.H. (2004). Production responses of weaned pigs after chronic exposure to airborne dust and ammonia. Anim. Sci..

[bib0140] Wellenberg G.J., Stockhofe-Zurwieden N., Boersma W.J., De Jong M.F., Elbers A.R. (2004). The presence of co-infections in pigs with clinical signs of PMWS in The Netherlands: a case–control study. Res. Vet. Sci..

[bib0145] Wieland B., Werling D., Nevel A., Rycroft A., Demmers T.G., Wathes C.M., Grierson S., Cook A.J., Done S.H., Armstrong D. (2012). Porcine circovirus type 2 infection before and during an outbreak of postweaning multisystemic wasting syndrome on a pig farm in the UK. Vet. Rec..

[bib0150] Young M.G., Cunningham G.L., Sanford S.E. (2011). Circovirus vaccination in pigs with subclinical porcine circovirus type 2 infection complicated by ileitis. J. Swine Health Prod..

