# Supplementary material for: Cost of post-weaning multi-systemic wasting syndrome and porcine circovirus type-2 subclinical infection in England – An economic disease model
Source: Prev Vet Med. 2013 Jun 1;110(2):88–102. doi: 10.1016/j.prevetmed.2013.02.010 (PMC3652492; doi:10.1016/j.prevetmed.2013.02.010)
Supplement: Supplementary file 1 [file mmc1.doc]

**Appendix 1.** Calculation of average daily weight gains and feed conversion rates for diseased pigs in the economic model.

The feed consumption rates and average daily weight gains were calculated the following way:

1. Feed conversion rate for a healthy pig (2.39 kg feed / kg weight gain) is based on the English Pig industry benchmarking data 2011. The value was obtained from the top third farms in the UK, which would more closely reflect the values of healthy pigs.
2. Reduction in feed consumption of diseased pigs was calculated using the results from the L-2001 study. In the study, food consumption was measured at pen level. The experiment followed nine batches over time. The pigs from each batch were separated in 5 rooms, and each room had 2 pens. After 41 days the mean average daily weight gain of pigs in a pen and the mean feed consumption per pig*day in a pen were calculated (pig*day was used as denominator to account for mortality). For the analysis only data from batches 8 and 9 were used, as these were the ones that suffered a PMWS outbreak. Results are shown in Figure 6. A linear regression on these values indicates a strong linear association between both variables (regression coefficient p-value<0.0001, R^2^=0.60).

**Figure 6.** Scatter plot of the pen level daily feed consumption rate (y axis) and pen level average daily weight gain, using data from batches 8 and 9 from the L-2001 study.

1. As explained in the manuscript, using the data from the L-2001 study, investigation was done to assess the differences in average daily gain (ADG) at animal level between (1) pigs not infected by PCV2 (mean ADG=0.424 kg.), (2) pigs infected by PCV2 but not showing PMWS clinical signs (mean ADG = 0.355 kg.), and (3) pigs infected by PCV2 displaying PMWS signs (mean ADG=0.313 kg.). Results obtained showed that pigs infected by PCV2 not showing PMWS signs had a 16% reduction in ADG compare to pigs not infected by PCV2 (p-value<0.001). Pigs infected by PCV2 displaying PMWS signs had a 26% reduction in ADG compare to not infected pigs (p<0.001).
2. Using the linear regression model explained in point 2, the difference in feed consumption between pens with different average daily weight gains was predicted. A pen in which pigs ADG equal to those of ‘PCV2 infected pigs not showing PMWS clinical signs’ (see point 3) had a 10% reduction in feed consumption compare to a pen with pigs having an ADG equal to ‘non-PCV2 infected pigs’. A 17% reduction in feed consumption was obtained between pigs not infected by PCV2 and pigs infected by PCV2 and showing PMWS clinical signs.
3. Base on these results the following parameters were used for the economic model:
   1. Reduction in Average daily gain of a PMWS pig during the clinical stage = 26%
   2. Reduction in Average daily gain of a Subclinical pig = 16%
   3. Appetite loss of a PMWS pig during the clinical stage = 17%
   4. Reduction in feed consumption of a Subclinical pig = 10% (this is to adjust the feed consumption to the lower weight gain of subclinical pigs)
4. When those parameters were applied, the following values were obtained in the model:
   1. Feed conversion rate of a Subclinical pig = 2.56
   2. Feed conversion rate of a PMWS-R pig = 2.60 (Note: these pigs when recovered have a FCR similar to a subclinical pig. If the pig remained in the clinical stage until slaughter, it would have had a FCR of 2.70)
5. Stochastic simulations were used to account for uncertainty in these parameters. Reduction in feed consumption was linked to the reduction in average daily gain of pigs in the stochastic model. For example, an increase in the ‘percentage reduction of ADG of a PMWS pig’ also means an increase in the appetite loss of this pig.
